# A New Oxaliplatin Resistance-Related Gene Signature With Strong Predicting Ability in Colon Cancer Identified by Comprehensive Profiling

**DOI:** 10.3389/fonc.2021.644956

**Published:** 2021-05-07

**Authors:** Qiu Lin, Li Luo, Hua Wang

**Affiliations:** Department of Colorectal Surgery, Affiliated Hospital of Integrated Traditional Chinese and Western Medicine, Nanjing University of Chinese Medicine, Nanjing, China

**Keywords:** oxaliplatin resistance, colon cancer, prognostic signature, LASSO, weighted gene co-expression network analyses

## Abstract

Numerous colon cancer cases are resistant to chemotherapy based on oxaliplatin and suffer from relapse. A number of survival- and prognosis-related biomarkers have been identified based on database mining for patients who develop drug resistance, but the single individual gene biomarker cannot attain high specificity and sensitivity in prognosis prediction. This work was conducted aiming to establish a new gene signature using oxaliplatin resistance-related genes to predict the prognosis for colon cancer. To this end, we downloaded gene expression profile data of cell lines that are resistant and not resistant to oxaliplatin from the Gene Expression Omnibus (GEO) database. Altogether, 495 oxaliplatin resistance-related genes were searched by weighted gene co-expression network analysis (WGCNA) and differential expression analysis. As suggested by functional analysis, the above genes were mostly enriched into cell adhesion and immune processes. Besides, a signature was built based on four oxaliplatin resistance-related genes selected from the training set to predict the overall survival (OS) by stepwise regression and least absolute shrinkage and selection operator (LASSO) Cox analysis. Relative to the low risk score group, the high risk score group had dismal OS (*P* < 0.0001). Moreover, the area under the curve (AUC) value regarding the 5-year OS was 0.72, indicating that the risk score was accurate in the prediction of OS for colon cancer patients (AUC >0.7). Additionally, multivariate Cox regression suggested that the signature constructed based on four oxaliplatin resistance-related genes predicted the prognosis for colon cancer cases [hazard ratio (HR), 2.77; 95% CI, 2.03–3.78; *P* < 0.001]. Finally, external test sets were utilized to further validate the stability and accuracy of oxaliplatin resistance-related gene signature for prognosis of colon cancer patients. To sum up, this study establishes a signature based on four oxaliplatin resistance-related genes for predicting the survival of colon cancer patients, which sheds more light on the mechanisms of oxaliplatin resistance and helps identify colon cancer cases with a dismal prognostic outcome.

## Introduction

Colon cancer is a frequently occurring gastrointestinal tract (GIT) cancer. As estimated by the International Agency for Research on Cancer (IARC, https://www.iarc.fr/), its morbidity and mortality rates in 2018 are 14.4 and 7.2%, separately, in the world. At present, colon cancer is mainly treated with surgery combined with chemotherapy (1, 2). Great progresses have been made in the early discovery and treatment of colon cancer; as a result, its morbidity and mortality rates decrease by 30–50% among the relapsed or metastatic cases in 5 years of treatment (3). Oxaliplatin is a kind of third-generation platinum-based anticancer drug and a diaminocyclohexane (DACH) platinum, which is now adopted for treating diverse cancers (4, 5). Certain cases can gain benefits from the oxaliplatin-based chemotherapy, yet others show no treatment response because they develop resistance to oxaliplatin (6–9). When used alone, only 20–24% of patients respond to oxaliplatin in the first-line treatment, and around 10% of refractory cases or those who fail in fluorouracil-based treatment respond to it in the second-line treatment (10). Therefore, it is urgently needed to manage oxaliplatin resistance, a cause of the poor survival of patients with advanced colon cancer. Consequently, it is of great significance to establish a signature based on oxaliplatin resistance- and prognosis-related genes for understanding the heterogeneities among colon cancers at the molecular level and improving treatment for these cases.

In recent years, researchers have carried out unsupervised cluster analysis on the transcriptomic data obtained from four colon cancer consensus molecular subtypes (CMSs). Among them, CMS1 stands for microsatellite unstable tumors (MSI) with great immune cell infiltration degree; CMS2 displays the activation patterns of the MYC and WNT pathways; tumors in CMS3 are featured by KRAS mutations as well as metabolic disorder; while CMS4 represents the mesenchymal subtype with epithelial–mesenchymal transition (EMT) and great stromal cell infiltration degree (11). Typically, the above CMS classification system facilitates to predict the patient prognosis, and cancers in CMS4 are linked to the poorest overall survival (OS) and relapse-free survival (RFS) (12). But this classification method cannot be used to estimate drug response in patients due to its oversimplification, so it is not quite helpful in individual treatment (13). Moreover, some biomarkers are utilized for predicting the prognosis of colon cancer patients. For instance, carbohydrate antigen 19-9 (CA19-9) has been identified as a predominant characteristic to diagnose colon cancer in clinical practice (14). CA19-9 is useful for the identification of outcomes of patients with stages I–III colorectal cancer 2 years after their operations (15). Unluckily, the CA19-9 level is neither discovered as the independent prognostic factor or the therapeutic target for improving patient OS nor used to predict patients' susceptibility to therapeutic drugs. Bioinformatic analysis has rapidly emerged as an important approach to assist the investigators in developing novel ideas regarding tumor research (16). More and more studies have recognized that mRNA expression signatures play an important role in predicting patient OS or relapse (17–19), yet no combined analysis is available to examine the relationship of the levels of oxaliplatin resistance-related genes with OS among colon cancer patients.

The present work downloaded the expression profile data of colon cancer cell lines resistant or sensitive to oxaliplatin from the Gene Expression Omnibus (GEO) database (20) and found oxaliplatin resistance-related genes by means of weighted gene co-expression network analysis (WGCNA) along with differential expression analysis. Besides, the mRNA expression pattern data of colon cancer patients in the training and test sets along with their clinical characteristics were analyzed to establish an mRNA-based model as the novel indicator for predicting patient prognosis. As a result, our constructed signature that integrated sufficient transcript data is helpful to risk stratification, which offered an approach to more accurately assess individual treatment for colon cancer cases. The above findings shed more light on the malignancy and treatment for individuals with colon cancer.

## Materials and Methods

### Data Extraction and Study Design

**Figure 1:**
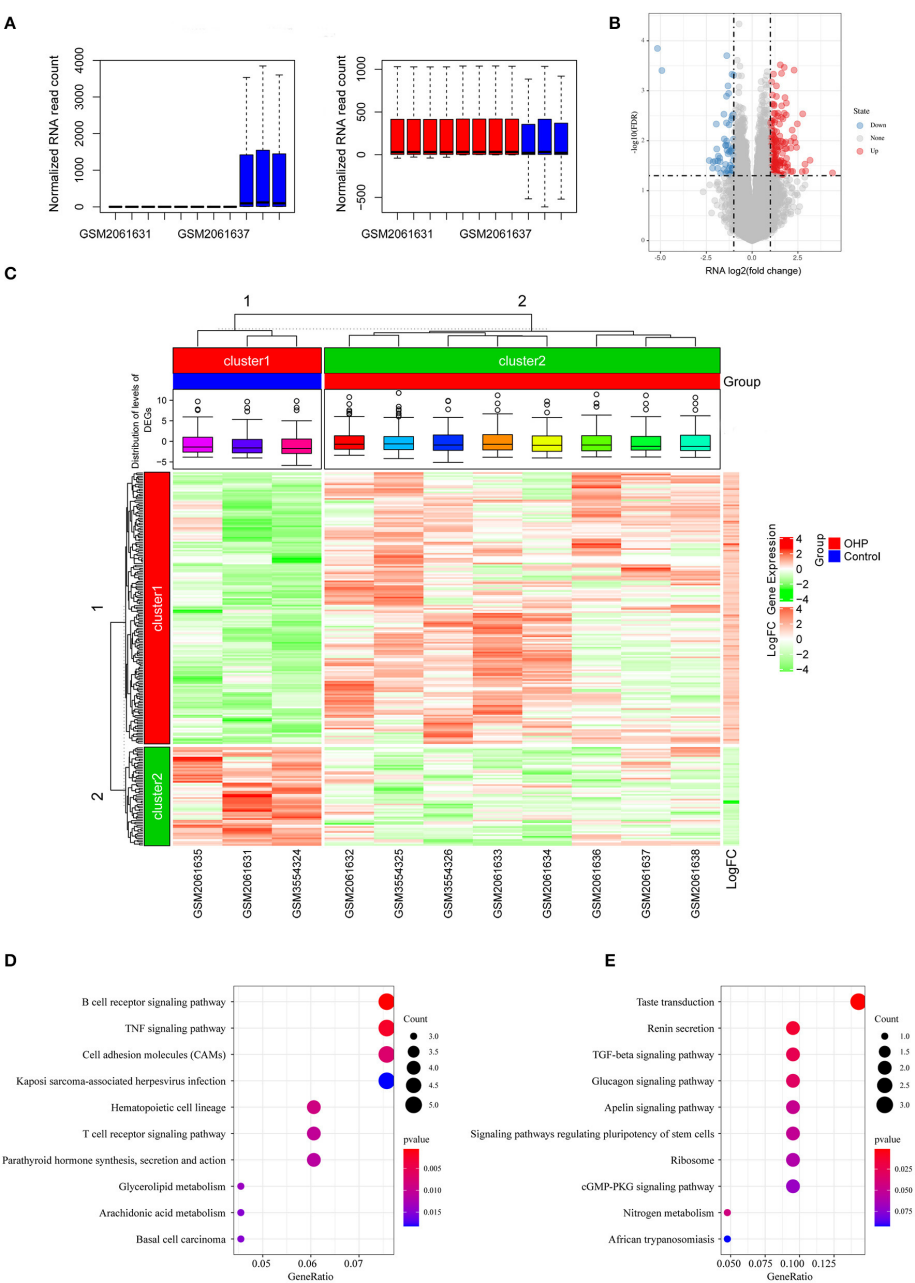
Differentially expressed genes (DEGs) between oxaliplatin-resistant and -sensitive colon cancer cells. **(A)** Boxplot of the distribution of the sample expression profile before and after the removal of the batch effect. **(B)** Volcanogram of DEGs. **(C)** Heatmap of DEGs. **(D)** Kyoto Encyclopedia of Genes and Genomes (KEGG) enrichment analysis of upregulated DEGs. **(E)** KEGG enrichment analysis of downregulated DEGs.

The expression profile data in oxaliplatin-resistant or non-resistant colon cancer cell lines were obtained from GSE77932 and GSE124808 datasets. The feature extraction software 10.7.3.1 (Agilent) was used for feature extraction, and the default parameters were used to analyze the scanned images, so as to obtain the processed signal intensity after background subtraction and spatial trend variation. The scanned data were imported by GeneSpring GX and normalized to 75%. Subsequently, the two datasets were merged and batch processed using the combat function of R package sva (Figure 1A), so as to obtain the gene expression matrix. Moreover, to construct the prognosis prediction model, we downloaded three datasets (including gene expression profiles, prognosis information, and other clinical features), namely, GSE17536, GSE17538 (21), and GSE39582 (22), with the first dataset as the training set and the latter two as the validation sets. All data used in this study were openly accessible, so there was no need to obtain approval from the ethics committee.

### Differentially Expressed Gene Analysis

We identified differentially expressed genes (DEGs) from colon cancer cells resistant (DLD1-derived oxaliplatin-resistant clones: DLD/OHP1, DLD/OHP4, and DLD/OHP5; HCT116-derived oxaliplatin-resistant clones: HCT/OHP1, HCT/OHP3, and HCT/OHP5) and sensitive (DLD1 and HCT116) to oxaliplatin by adopting limma package (23) in line with the selection thresholds of false discovery rate (FDR) <0.05, |log2 fold change (FC)| >1, and *P* < 0.05. DEGs that satisfied the above thresholds were screened in subsequent analysis. In addition, the limma and pheatmap packages were utilized to draw the volcano plot and heatmap separately.

### Identification of Oxaliplatin Resistance-Related Genes by Weighted Gene Co-expression Network Analysis

We constructed a co-expression network that targeted oxaliplatin resistance using the WGCNA package (24). Firstly, we carried out cluster analysis on the samples by hierarchical clustering. A weight co-expression network was constructed using the WGCNA of R package, and the soft threshold was set at eight to select the co-expression modules. Later, we confirmed that our established co-expression network was consistent with a scale-free network. In other words, the node/k connectivity [log(k)] logarithm showed a negative correlation with the node [log(P(k)] occurrence probability logarithm, with the coefficient of correlation being >0.8. To guarantee the scale-free network, the β value was set at eight. Later, we transformed the expression matrix to the close matrix and transformed it to the topological matrix to carry out gene clustering based on TOM by adopting the average linkage hierarchical cluster method according to the standards of mixed dynamic shear tree. Besides, over 30 genes were screened for each gene network module. Gene modules were determined by the dynamic shear methods, then the value of each module eigengene was determined successively, modules were subjected to cluster analysis, and the close modules were combined for forming a new module. Thereafter, we also determined the relationships between the gene modules identified and oxaliplatin resistance to mine the substantially associated gene modules for subsequent analysis.

### Pathway Enrichment Analysis

Kyoto Encyclopedia of Genes and Genomes (KEGG) analysis was conducted using the clusterProfiler package (25) for exploring and determining the possible biological functions of all critical genes. The significance levels were FDR <0.05 and *P* < 0.05. We employed the R software to draw the bubble plot for result visualization.

### Establishment of the Protein–Protein Interaction Network and Topological Analysis

We utilized the Search Tool for the Retrieval of Interacting Genes (STRING) online approach (26) to construct the protein–protein interaction (PPI) network of critical genes. As observed from the graph, all nodes within the network were greatly connected to each other. Then, the topological characters of nodes in the network were further analyzed, and the Degree, Betweenness centrality, Closeness centrality, and Eigenvector centrality values were calculated. In this study, genes in the PPI network that had all parameters greater than or equal to the medians of all nodes were considered to exert core roles in the network (in other words, the key oxaliplatin resistance-related prognostic genes), which were used to construct the subsequent prognosis model of colon cancer.

### Establishment of a Risk Assessment Model

The survival and glmnet packages were utilized to select the most appropriate genes that might be used to construct a model by least absolute shrinkage and selection operator (LASSO) Cox regression analysis. Typically, LASSO regression can be used to select variables to fit the high-dimensional generalized linear model. In this study, LASSO regression was conducted to construct a penalty function, which facilitated to obtain the improved model with a reduced number of variables and might avoid overfitting. We employed the glmnet package for determining the penalty parameter lambda through cross-validation; besides, we discovered the best lambda value associated with the lowest error mean of cross-validation. Thereafter, we selected the optimal gene group (lambda = 0.0508) for subsequent model construction. Furthermore, based on the expression profiles of feature genes, we utilized the stepAIC method in MASS package for stepwise multivariate regression analysis. Starting from the most complicated model, we deleted one variable each time in succession to reduce the Akaike Information Criteria (AIC) value (a smaller value indicated the better model, suggesting that the model utilized less parameters to obtain enough degree of fitting). Later, gene sets with the most appropriate AIC value were selected to construct the colon cancer risk prediction model. Then, risk scores were calculated after linearly combining the results of each coefficient determined by LASSO Cox regression multiplied according to the respective gene level and categorized the patients into the high-risk or low-risk groups. Besides, multivariate Cox regression was conducted to analyze whether the risk model was able to independently predict prognosis.

### Gene Set Enrichment Analysis

To observe the relationships between risk score and signal pathways, we selected the corresponding gene expression profiles in training set samples for single-sample gene set enrichment analysis (GSEA) using the GSVA function of R package (27) and calculated the scores of each sample in different signal pathways (in other words, we obtained the ssGSEA score of each sample in the corresponding pathway). Furthermore, we determined the correlations of these scores with risk score and selected FDR <0.5 as the criterion to judge pathways significantly related to the risk score.

### Statistical Analysis

The R software was employed for statistical analysis. In this study, data were expressed as medians. Log-rank test and Kaplan–Meier method were used to analyze the difference in OS between high and low risk score groups. In addition, the Cox proportional hazard regression model was used for univariate as well as multivariate analysis. The merge script in Perl language was utilized for data set merging. A difference of *P* < 0.05 indicated statistical significance.

## Results

### Differentially Expressed Genes Between Oxaliplatin-Resistant and -Sensitive Colon Cancer Cells and Pathway Enrichment

In this study, we downloaded the GSE77932 and GSE124808 gene sets from the GEO database to obtain gene expression profiles. As observed from the volcano plot (Figure 1B, Supplementary Table 1), there was differential mRNA expression in oxaliplatin-resistant colon cancer cells compared with the oxaliplatin-sensitive cells. Relative to the sensitive group, we obtained altogether 229 DEGs in resistant groups, among which 168 were upregulated while 61 were downregulated (*P* < 0.05). We also drew the heatmap to display the significant DEGs (Figure 1C). Thereafter, KEGG pathway enrichment was conducted on DEGs by using clusterProfiler package, which revealed that the upregulated genes were mainly enriched into the B cell receptor signaling pathway and tumor necrosis factor (TNF) signaling pathway, whereas the downregulated genes were mainly enriched into the transforming growth factor (TGF)-beta signaling pathway, Ribosome and cGMP-PKG signaling pathway (Figures 1D,E).

### Selection of Oxaliplatin Resistance-Related Gene Modules Through Weighted Gene Co-expression Network Analysis

**Figure 2:**
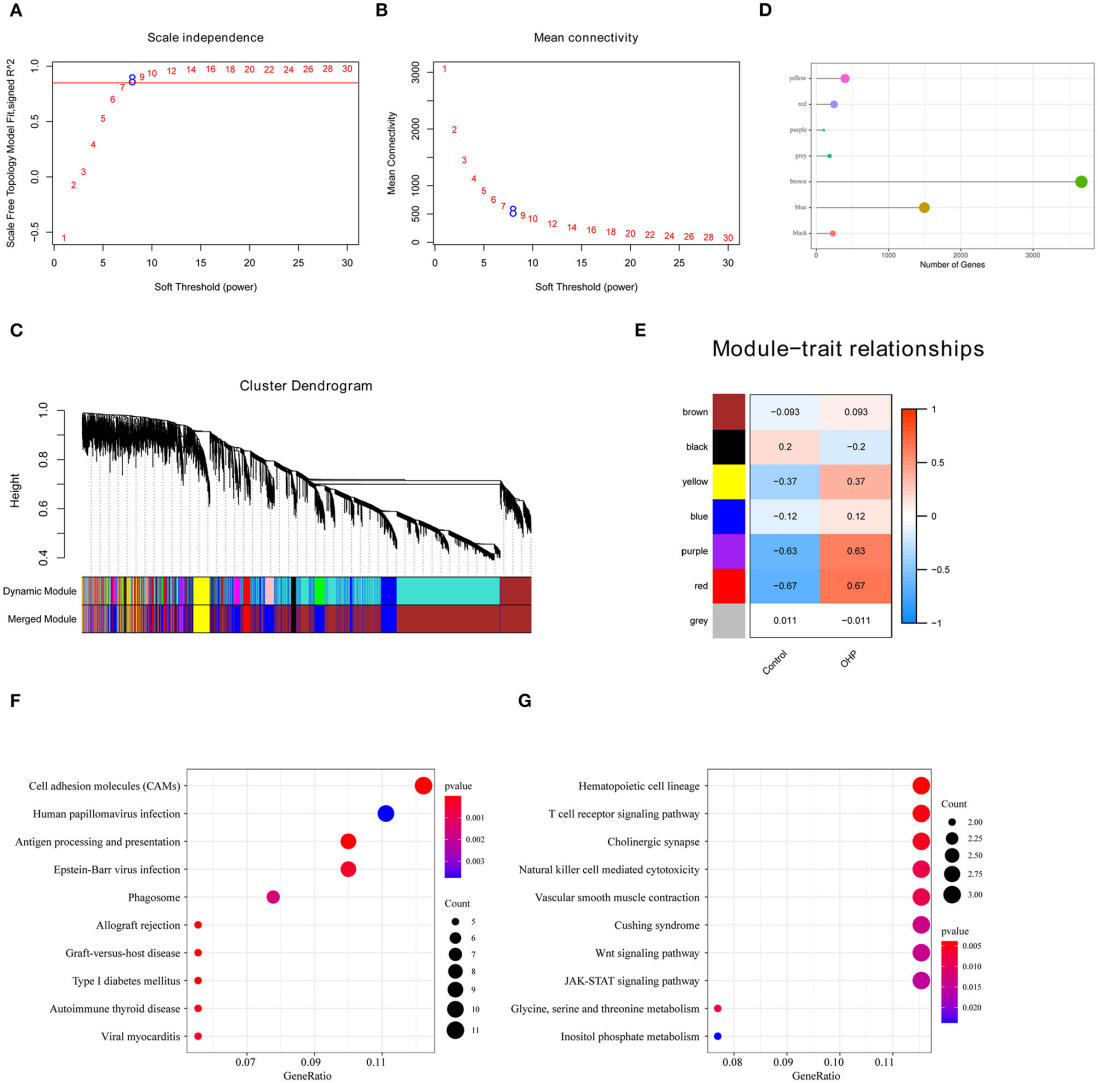
Oxaliplatin resistance-related gene modules mined through weighted gene co-expression network analysis (WGCNA). **(A,B)** Analysis of network topology for various soft-thresholding powers. **(A)** Analysis of the scale-free fit index for various soft-thresholding powers (β). **(B)** Analysis of the mean connectivity for various soft-thresholding powers. **(C)** Gene dendrogram and module colors. **(D)** The number of genes in each module. **(E)** Correlation between each module and oxaliplatin resistance events. **(F)** The Kyoto Encyclopedia of Genes and Genomes (KEGG) enrichment analysis of the genes in the red module. **(G)** The KEGG enrichment analysis of the genes in the purple module.

Apart from screening DEGs between oxaliplatin-resistant and -sensitive colon cancer cells, we also established the gene co-expression network to identify gene modules with biological significance through WGCNA and to better discover the significant oxaliplatin resistance-related genes in the context of colon cancer. We acquired altogether seven modules in later analysis (Figures 2A–D). Subsequently, we analyzed the correlations of these gene modules with the oxaliplatin resistance events and discovered that the red and purple modules were most significantly correlated with resistance (Figure 2E). Later, those genes in the red and purple modules were subjected to KEGG pathway enrichment by using the clusterProfiler package. As a result, genes in the red module were mainly enriched into Autoimmune thyroid disease, Phagosome, and Cell adhesion molecules (CAMs) pathways; whereas genes in the purple module were mainly enriched into the JAK-STAT, Wnt, and T cell receptor signal pathways (Figures 2F,G), demonstrating that the resistance of colon cancer to oxaliplatin might be related to immunity and cell migration.

### Identification of Key Oxaliplatin Resistance-Related Genes for Predicting the Prognosis of Colon Cancer

**Figure 3:**
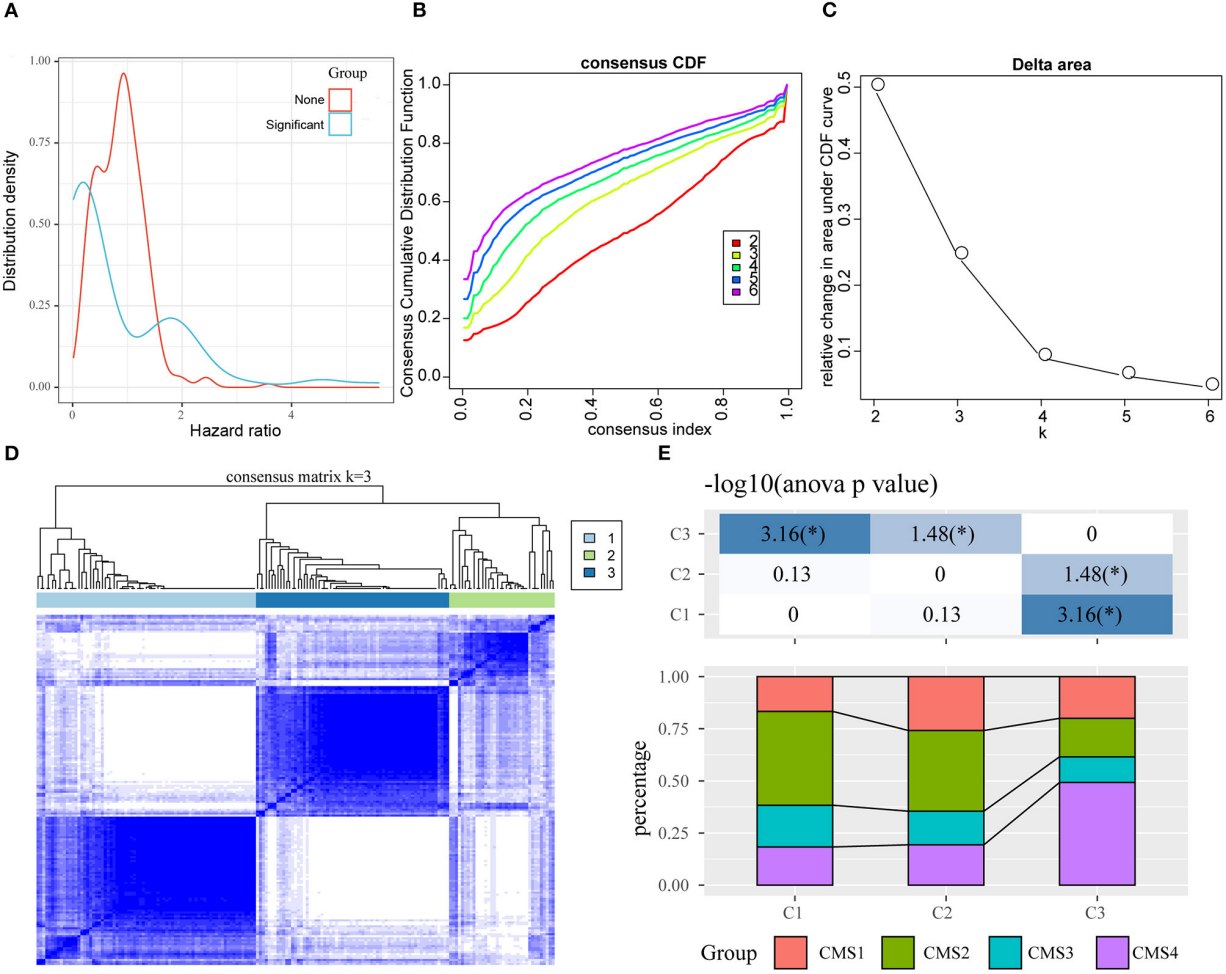
Identification of oxaliplatin resistance-associated subtypes of colon cancer in the training set. **(A)** The hazard ratio (HR) distribution of genes related to oxaliplatin resistance, wherein “Sig” represents the HR distribution of genes significantly related to prognosis and “None” represents the HR distribution of genes not significantly related to prognosis. **(B)** The cumulative distribution function (CDF) curves of consensus scores based on different subtype numbers (k = 2~6) and the corresponding color are represented, which could help us determine the choice of k when the CDF reaches the maximum (aiming to reach the maximal consistency and cluster confidence). **(C)** The comparison of the relative changes of the area under the CDF curve between k and k−1, which can help users determine the relative increase in consensus and the value of k with significant increase. **(D)** The consensus score matrix of colon cancer samples when k = 3 (1 = C1, 2 = C2, 3 = C3). **(E)** The distribution of samples from the three subtypes in the CMS subgroups.

First of all, we had integrated 394 genes in the oxaliplatin resistant-related gene modules (red and purple), with 229 DEGs in between oxaliplatin-sensitive and -resistant colon cancer cell lines. Finally, we acquired 495 genes after removing duplication. Secondly, using univariate survival analysis, we determined the relationships between the expression of these 495 genes and prognosis based on the prognosis data (training set, GSE17536). By adopting the threshold of *P* < 0.05, we obtained 79 genes that showed distinct OS (Figure 3A, Supplementary Table 2). Furthermore, we used these genes for sample consistent clustering using the R package ConsensusClusterPlus (V1.48.0; parameters: reps = 100, pItem = 0.8, pFeature = 1, and distance = “spearman”). D2 and Euclidean distance (ED) were used as the clustering algorithm and distance metric, respectively. At k = 2–6, the samples were clustered into three clusters (C1, C2, C3; Figures 3B–D). We compared the relationships between C1–3 samples and the previous published CMS classification samples. As shown in Figure 3E, we discovered that samples in the C3 subtype were mainly enriched into the CMS4 subtype, while those in the C1 subtype were significantly enriched in the CMS2 subtype. In CMS classification, CMS4 had the poorest prognosis, while CMS2 had the best prognosis. This revealed that the expression profiles of these 79 prognosis-based oxaliplatin resistance-related genes were able to classify patient prognosis to some extent.

**Figure 4:**
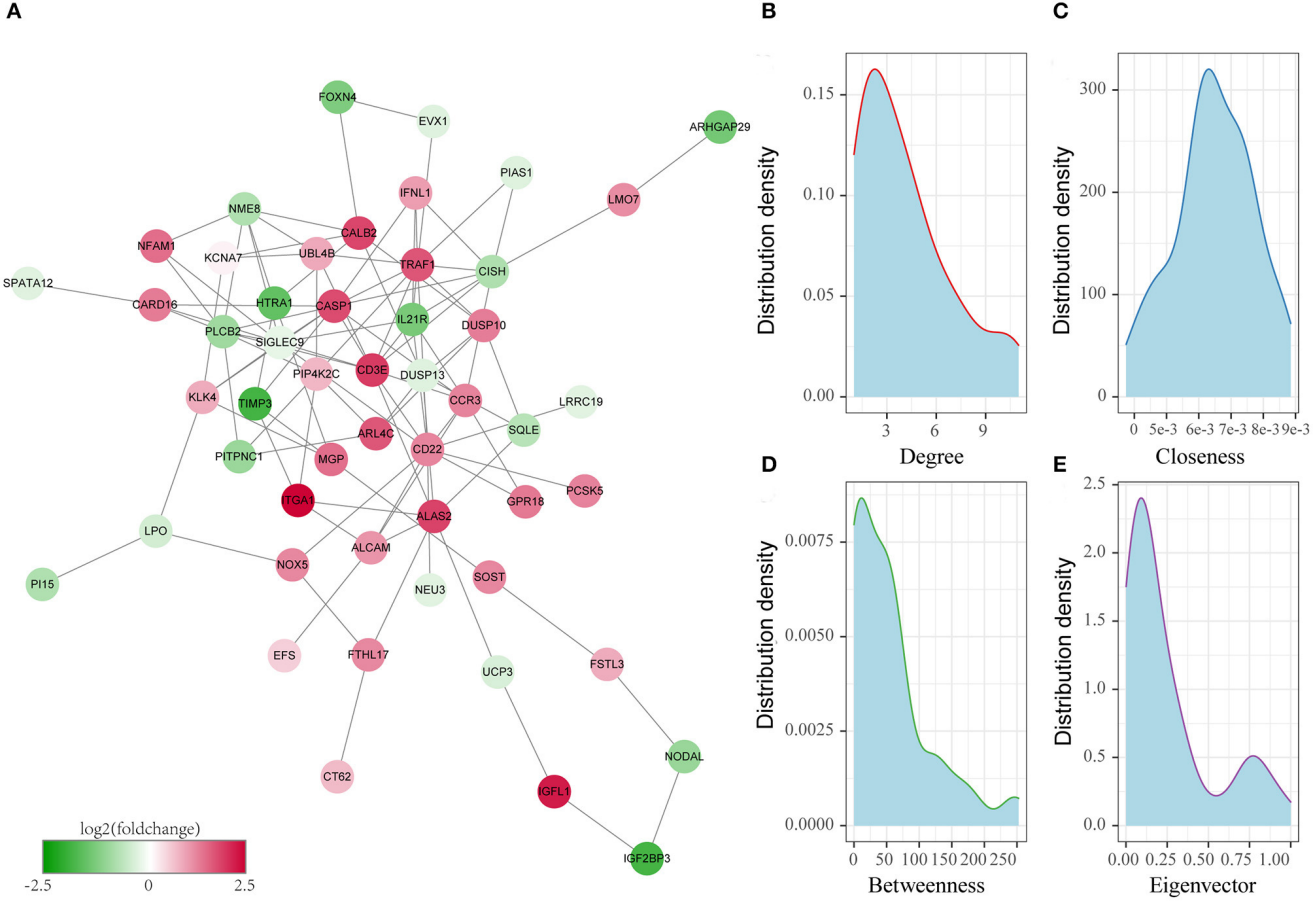
Identification of the key oxaliplatin resistance-related genes for predicting the prognosis of colon cancer. **(A)** Protein–protein interaction (PPI) network of prognostic genes related to oxaliplatin resistance. **(B)** Degree distribution of genes in the network. **(C)** Closeness centrality distribution of genes in the network. **(D)** Betweenness centrality distribution of genes in the network. **(E)** Eigenvector centrality distribution of genes in the network.

**Figure 5:**
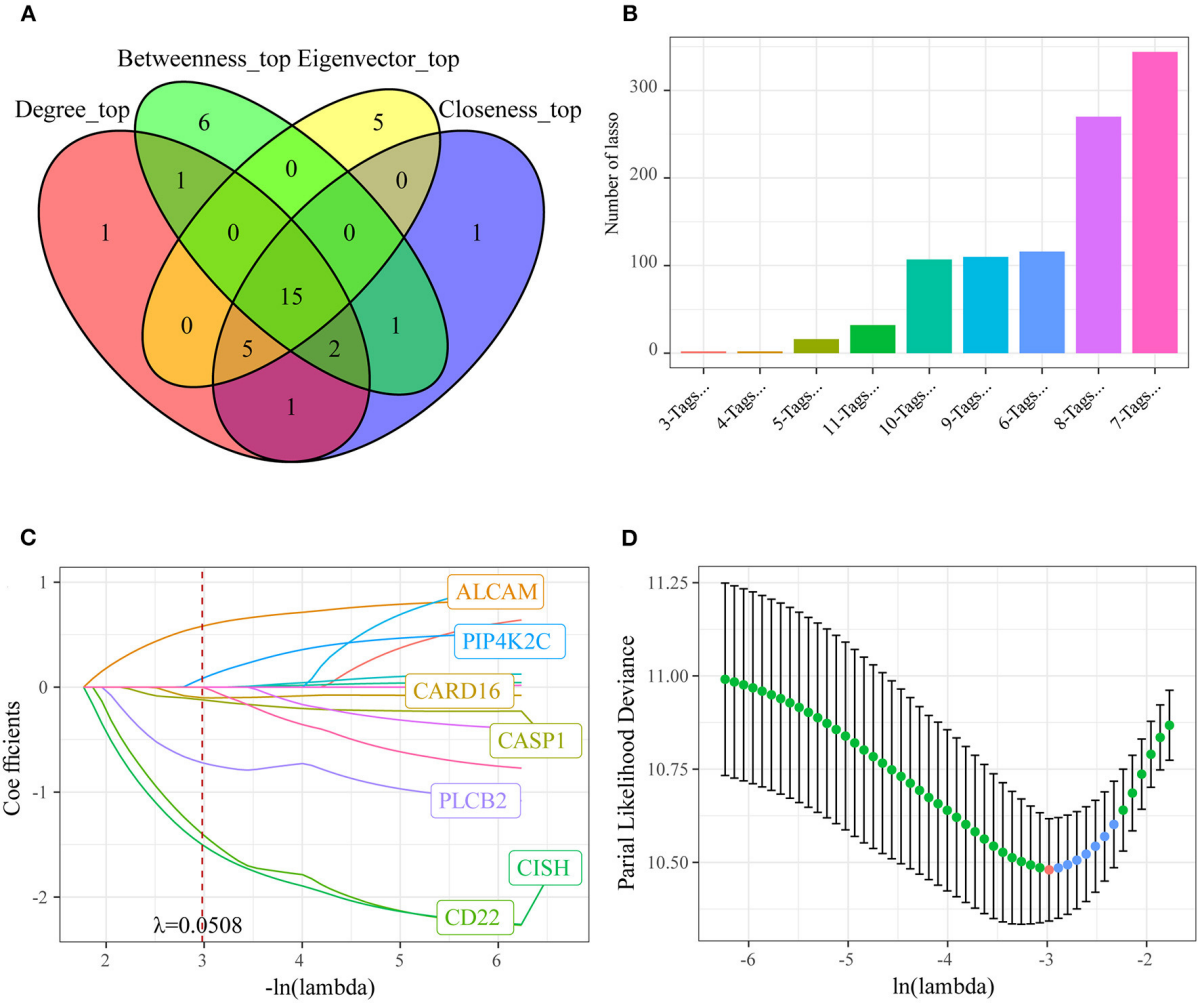
Identifying the prognosis signature related to oxaliplatin resistance for colon cancer by least absolute shrinkage and selection operator (LASSO). **(A)** Fifteen genes whose values of all the above four parameters were greater than or equal to the median of all nodes were selected as the key prognostic markers related to the resistance of colon cancer to oxaliplatin. **(B)** Frequency of different gene combinations in a thousand times of LASSO regressions. **(C)** The changing trajectory of each independent variable. The horizontal axis represents the log value of the independent variable lambda, and the vertical axis represents the coefficient of the independent variable. When lambda = 0.0508, the coefficients were not 0 in seven genes. **(D)** Confidence intervals for each lambda. We chose the lambda with the smallest average standard deviation as the optimal model, that is, lambda = 0.0508.

Thirdly, to further screen the key prognosis genes, we mapped these 79 genes to the STRING database to obtain the protein interaction networks of these genes. As shown in Figure 4A, 51 of these 79 genes showed interactions. Subsequently, we analyzed the topological properties of the nodes in the network and calculated degree, Closeness centrality, Betweenness centrality, and Eigenvector centrality (Supplementary Table 3). We discovered that the degree of nodes in the network was mainly 1–4 (Figure 4B), the closeness centrality was 0.005–0.008 (Figure 4C), the betweenness centrality was 0–100 (Figure 4D), and the Eigenvector centrality was 0–0.25 (Figure 4E). In addition, we also found that there were fewer nodes with higher levels of topological parameters and more nodes with lower ones in the network, which showed a power-law distribution and was in line with the characteristics of biological network. Finally, 15 genes (Figure 5A) whose values of all the above four parameters were greater than or equal to the median of all nodes were selected as the key prognostic markers related to the resistance of colon cancer to oxaliplatin, which were used for further analysis and prognosis model construction.

### Establishment of the Oxaliplatin Resistance-Related Risk Assessment Model

To investigate the effect of those screened genes in predicting the prognosis of colon cancer, we incorporated 15 key genes into LASSO and stepwise regression for identifying the potent markers. Thereafter, we established a prognosis signature based on four genes [CD22, CASP1, CISH, and activated leukocyte cell adhesion molecule (ALCAM)] to evaluate the prognosis for colon cancer patients (Figures 5B–D). Besides, the risk score of every colon cancer case from the training set was determined based on the four gene coefficients.

$$\begin{array}{l} \text{Risk score }=-2.889^{*}CD\text{22}-0.323^{*}CASP\text{1}-2.23^{*}CISH\\ \;+0.816^{*}ALCAM. \end{array}$$

**Figure 6:**
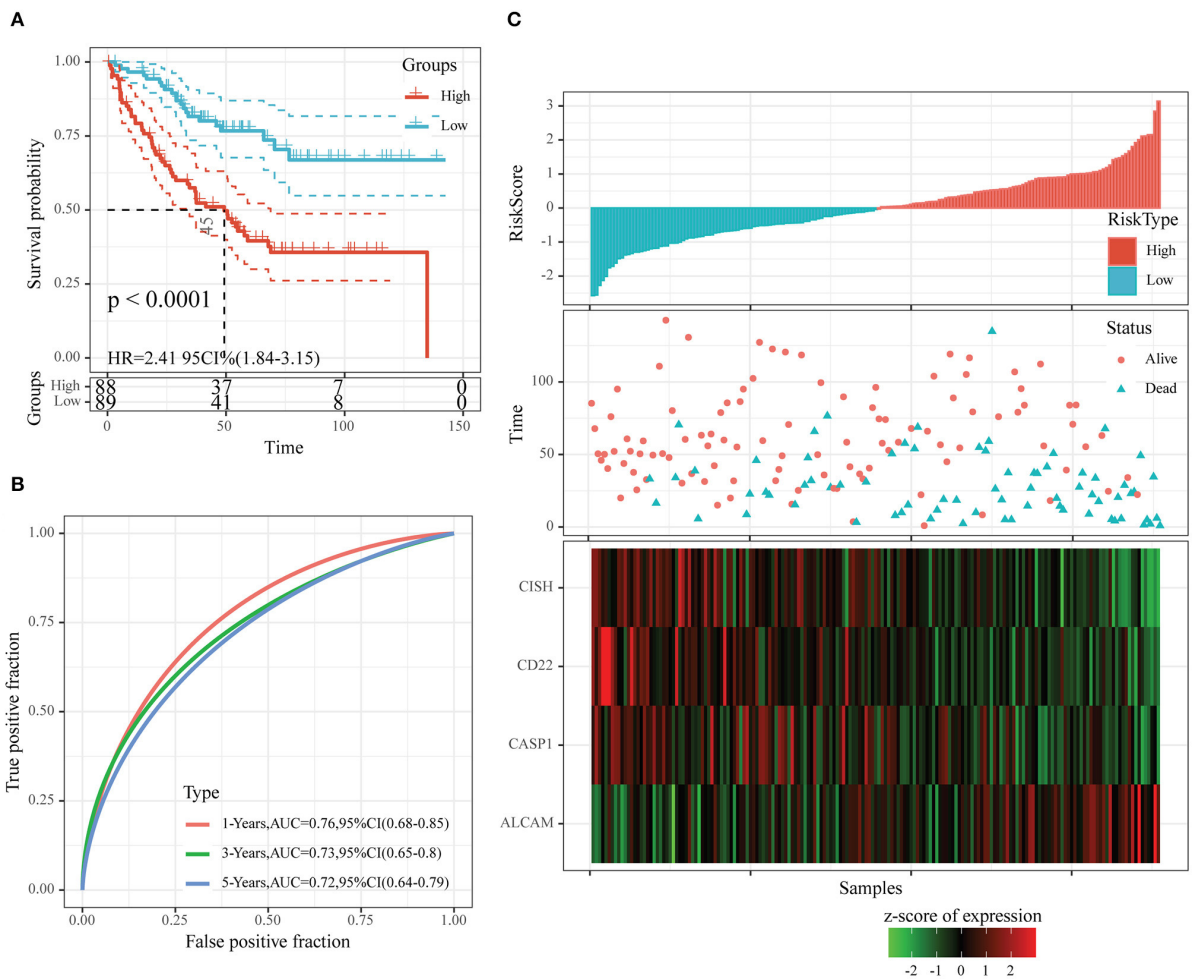
The classification effect of the four-oxaliplatin resistance-related gene-based prognosis signature in the training set. **(A)** Analyzed the prognostic difference after predicted classification according to the four-gene signature in the training set. **(B)** Receiver operating characteristic (ROC) curves of the four-gene signature in colon cancer samples in the training set. **(C)** The relationship of risk score, survival time, and survival status with the expression levels of the four genes in the training set.

After determining the risk scores of all samples in the training set, we divided all samples into high- or low-risk group in line with the median risk score (cutoff = 0). Figure 6 displays the classification accuracy of our constructed prognosis model for training set samples. It was illustrated from Figure 6A that 89 and 88 cases were divided as the low- and high-risk groups, respectively, and the difference in prognosis was statistically significant between both groups (*P* < 0.0001, HR = 2.41, 95% CI = 1.84–3.15). Figure 6B displays the receiver operating characteristic (ROC) curves. As observed, the AUC values at 1, 3, and 5 years, respectively, were 0.76, 0.73, and 0.72. Besides, Figure 6C revealed that the dead samples had evidently reduced survival time as the risk score increased, and more dead samples were observed in the high-risk group. Moreover, high ALCAM expression was recognized as a risk factor on the basis of changes in the levels of those four prognostic genes as risk score elevated. In comparison, high CD22, CISH, and CASP1 expression was associated with a decreased risk, and they served as the protective factors.

### Validation of the Robustness of Our Four-Oxaliplatin Resistance-Related Gene-Based Prognosis Signature

**Figure 7:**
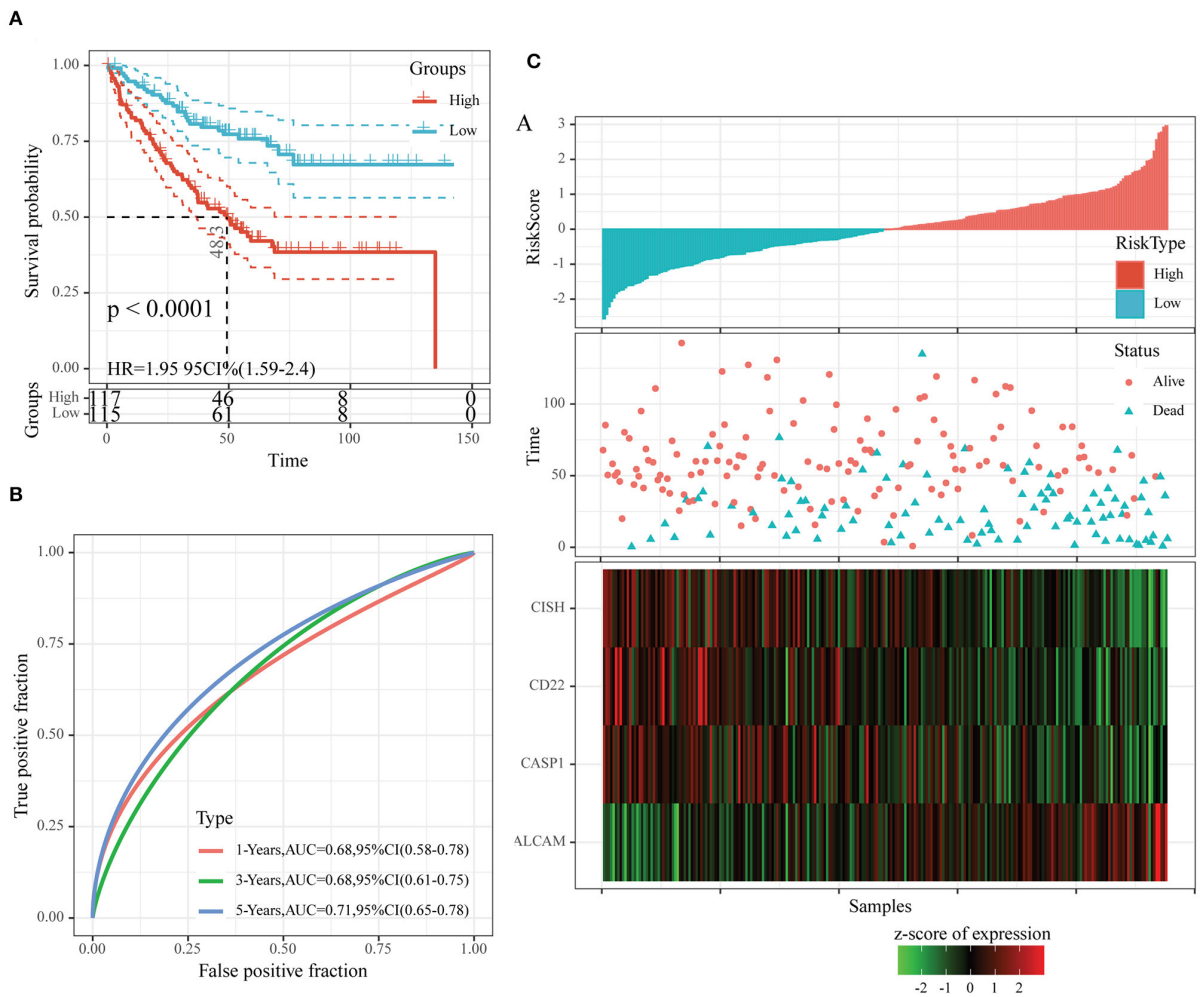
The classification effect of the four-oxaliplatin resistance-related gene-based prognosis signature in the first test set (GSE17538). **(A)** Analyzed the prognostic difference after predicted classification according to the four-gene signature in the first test set (GSE17538). **(B)** Receiver operating characteristic (ROC) curves of the four-gene signature in colon cancer samples in the first test set (GSE17538). **(C)** The relationship of risk score, survival time, and survival status with the expression levels of the four genes in the first test set (GSE17538).

To examine the robustness of our constructed four-gene model, we used the same model and threshold as those in the training set for verification in the test set. Figure 7 displays the classification accuracy in the first test set (GSE17538). As observed from Figure 7A, 115 and 117 cases were divided as low- and high-risk groups, respectively, and there was a significant difference in prognosis between them (*P* < 0.0001, HR = 1.95, 95% CI = 1.59–2.40). Figure 7B exhibits the ROC curves, with the AUC values at 1, 3, and 5 years, respectively, being 0.68, 0.68, and 0.71. Figure 7C shows similar findings to those obtained from the training set. In other words, dead samples had markedly shorter survival time as the risk score increased, and more dead samples were observed in the high-risk group. In addition, high ALCAM expression was a risk factor, while high CD22, CISH, and CASP1 levels were the protective factors.

**Figure 8:**
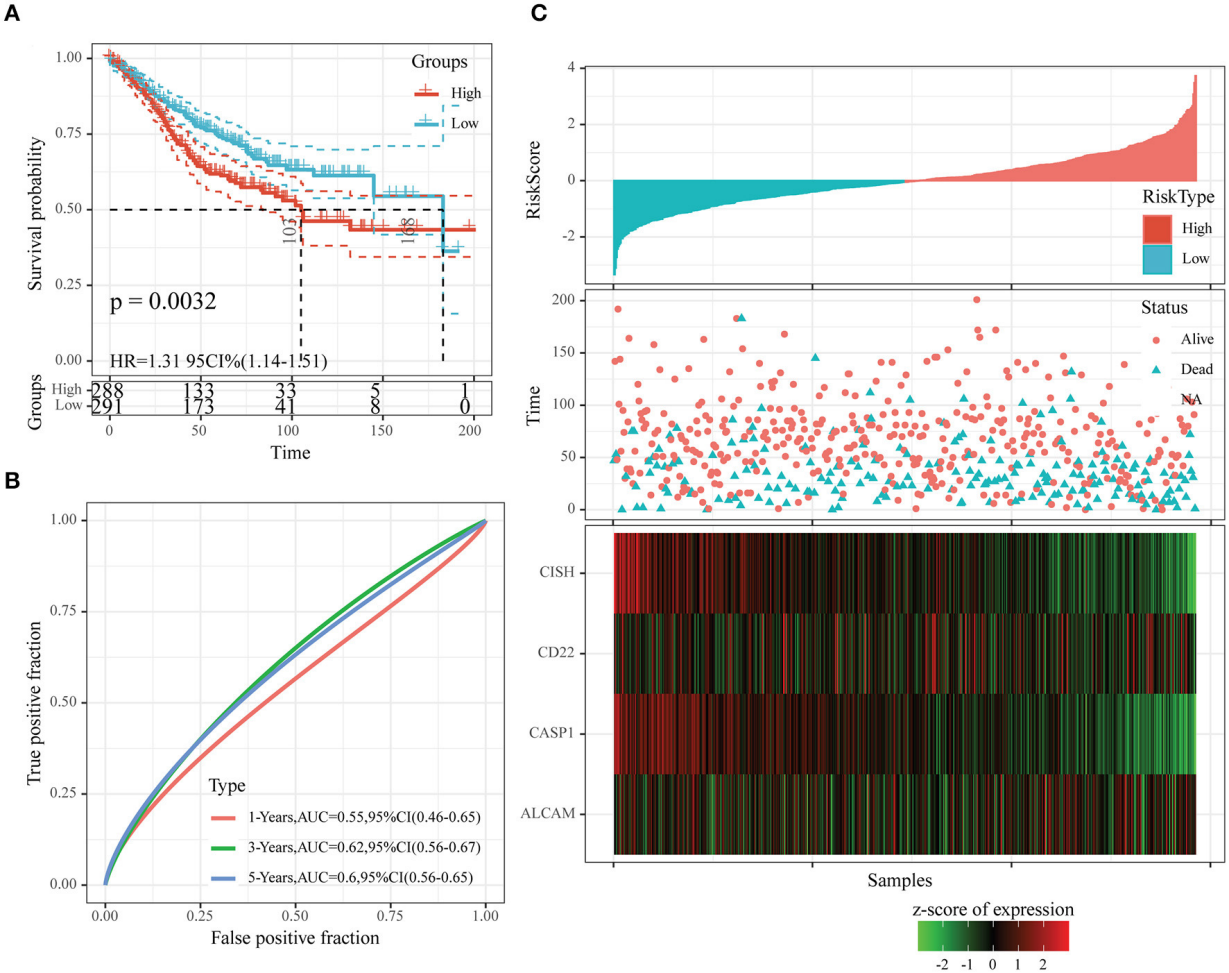
The classification effect of the four-oxaliplatin resistance-related gene-based prognosis signature in the second test set (GSE39582). **(A)** Analyzed the prognostic difference after predicted classification according to the four-gene signature in the second test set (GSE39582). **(B)** Receiver operating characteristic (ROC) curves of the four-gene signature in colon cancer samples in the second test set (GSE39582). **(C)** The relationship of risk score, survival time, and survival status with the expression levels of the four genes in the second test set (GSE39582).

Moreover, we also downloaded the GSE39582 dataset (the second test set) from the GEO database as an external dataset and determined the risk scores for all samples by our model. We also used the threshold in the training set for classifying samples into high- or low-risk group. According to Figure 8A, the prognosis in the low-risk group was superior to that in the high-risk group. Upon ROC analysis, comparable AUC values at 1–5 years to those of the first test set (GSE17538) and training set were obtained (Figure 8B). Furthermore, the associations of those four gene expression levels with risk score were the same as those obtained from the other two datasets (Figure 8C). In conclusion, our prognosis model constructed based on four oxaliplatin resistance-related genes performed well in predicting the prognosis for colon cancer.

### Clinical Independence of Our Constructed Signature Based on Four Oxaliplatin Resistance-Related Genes

**Figure 9:**
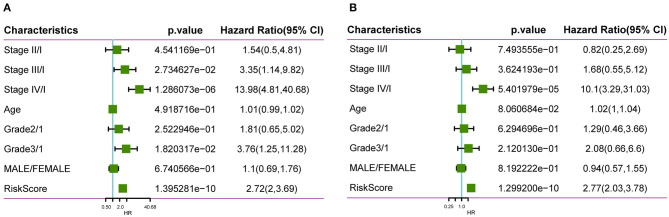
Clinical independence of our constructed signature based on four oxaliplatin resistance-related genes. Univariate **(A)** as well as multivariate **(B)** Cox regression analysis on clinical data and risk score from the training set was employed to calculate the corresponding HRs, 95% CIs, and *P*-values.

To identify whether our constructed model based on four oxaliplatin resistance-related genes was independent in clinical practice, we employed univariate as well as multivariate Cox regression analysis on clinical data and risk score from the training set and calculated the corresponding HRs, 95% CIs, and *P*-values. Figure 9 displays the grouping data of the four-gene signature. Univariate analysis was conducted on training set samples, which suggested that a high risk score, tumor stage III/IV, and Grade 3 showed marked correlations with dismal prognosis, but multivariate analysis discovered that only a high risk score (HR = 2.77, 95% CI = 2.03–3.78, *P* = 1.3e^−10^) as well as tumor stage IV (HR = 10.11, 95% CI = 3.29–31.03, *P* = 5.4e^−5^) displayed clinical independence. As a result, the prognosis signature constructed based on the four oxaliplatin resistance-related genes might serve as an independent prognostic indicator to predict patient prognosis clinically for colon cancer patients.

### Differences in Pathways Enriched Between High- and Low-Risk Groups Detected by Gene Set Enrichment Analysis

**Figure 10:**
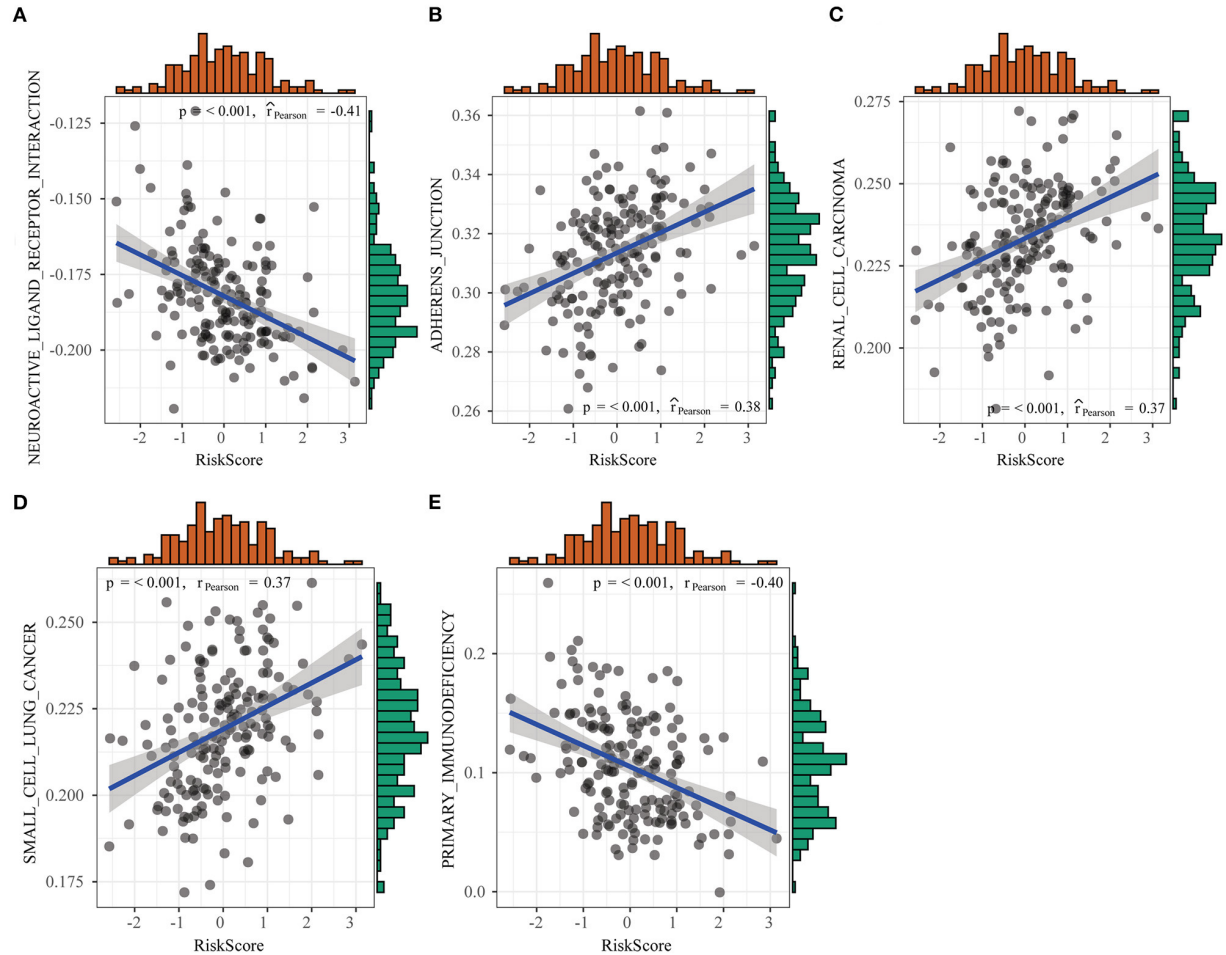
Differences in pathways enriched between high- and low-risk groups detected by gene set enrichment analysis (GSEA).

In this study, we used GSEA to examine pathways that were significantly enriched into both groups in the training set and obtained altogether five significantly enriched pathways (Figure 10), which included pathways tightly related to tumor occurrence, immunity, and metastasis, such as primary immunodeficiency, adherens junction, as well as pathways in cancer. Such result further verified that the resistance and poor prognosis of colon cancer were related to cancer cell migration and the immunosuppression status in the microenvironment.

## Discussion

Colon cancer is one of the most common cancers and remains one of the leading causes of cancer death worldwide (28). Colon cancer represents a complicated disorder that has numerous risk factors, including lifestyle, dietary habit, and genetics (29). Typically, colon cancer is usually featured by its intra-cancer heterogeneity; as a result, each patient is different with regard to the clinical presentations as well as treatment response (30). Therefore, it is necessary to tailor the treatment for colon cancer on the basis of risk and genetic factors of an individual. At present, the oxaliplatin-based chemotherapy is adopted in combination with radical surgery as the standard treatment for cases with colon cancer. Patients respond well to the first treatment, but many of them may experience resistance to oxaliplatin and subsequently develop recurrence, resulting in a dismal prognostic outcome (31, 32). Currently, the Tumor, Lymph node, Metastasis (TNM) classification system is widely utilized as the standard to predict relapse among colon cancer cases (33). However, it cannot achieve satisfactory performance in predicting prognosis and managing colon cancer. As a result, many researchers are devoted to developing novel strategies to improve the predicting accuracy of oxaliplatin resistance and patient prognosis and contribute to decision-making for individuals based on molecular biomarkers and clinicopathological features (34, 35).

Some single genes have been suggested as potent approaches to assess colon cancer prognosis. For instance, certain works emphasize on the significance of Wnt5a and Immature Colon Carcinoma Transcript-1 (ICT1) in prognosis prediction (36, 37). Similarly, one article focuses on the significance of the prediction model constructed based on five transcriptional factors in predicting prognosis. This model is constructed using the Cox PH model based on analysis of The Cancer Genome Atlas (TCGA)-derived colon cancer patients by the random forest algorithm (38). Some encouraging results have been discovered, but there is no biomarker available clinically to predict the prognosis for colon cancer. In addition, these prognostic genes and multi-gene signatures have not been identified as prediction factors of a response to chemotherapy in colon cancer. Furthermore, the joint action of several oxaliplatin resistance-related genes on colon cancer prognosis has not been investigated in a large-scale genomic study so far. As a result, it is of great importance to construct a novel model to predict the outcome of oxaliplatin-resistant colon cancer, which may also facilitate the prognosis prediction for colon cancer cases and decision of treatment strategy.

Consequently, this study aimed to establish a model based on oxaliplatin resistance-related genes to estimate the OS of colon cancer cases. In this work, we constructed a candidate risk model based on oxaliplatin resistance-related genes to estimate the survival of colon cancer cases based on results of the WGCNA, differential expression analysis, and Cox proportional regression analysis. The risk scores calculated based on the expression levels and coefficients of four mRNAs (ALCAM, CD22, CASP1, and CISH) might be used to precisely and independently predict the prognosis of colon cancer. First of all, altogether, 229 DEGs were identified between colon cancer cells that were resistant and sensitive to oxaliplatin. Secondly, 394 genes were screened from the oxaliplatin resistance-related gene modules on the basis of WGCNA. To explore the biological functions of these screened genes, we carried out KEGG pathway enrichment, which suggested that the above genes were mostly enriched into numerous drug resistance-related pathways, including cell adhesion, JAK-STAT, immune-related pathways, TGF-beta, and Wnt. Thirdly, univariate analysis and PPI network topological analysis were performed to determine the significant oxaliplatin resistance-related genes that might be used to predict the prognosis for colon cancer. Afterward, we established a model to predict the prognosis of colon cancer using four oxaliplatin resistance-related genes (ALCAM, CD22, CASP1, and CISH) on the basis of stepwise regression and LASSO Cox regression. Typically, CD22, CISH, and CASP1 were identified to be the independent protective factors, whereas ALCAM as the risk factor. It was surprising that according to ROC curve and survival analyses, the prognosis model-produced risk score might be utilized to be an accurate OS indicator for colon cancer. Besides, we compared our new risk score with the traditional clinicopathological factors, which verified that the prognosis effect was independent. At last, we adopted GSEA to analyze those pathways markedly enriched in the high- or low-risk group and detected five pathways showing significant differential enrichment between the two groups, including pathways that were tightly related to tumor occurrence, metastasis, and immunity, such as primary immunodeficiency, adherens junction, as well as pathways in cancer. The above preliminary results shed new light on the development of markers based on oxaliplatin resistance genes to predict the prognosis of colon cancer. Our proposed risk score may offer a novel direction to evaluate the prognosis for colon cancer, and it is distinct from the conventional evaluation system. It can help to further stratify patients, thus contributing to designing individual treatment and improving patient survival.

Of the screened four genes (ALCAM, CD22, CASP1, and CISH), CD22 is a sialic acid-binding immunoglobulin-like lectin (Siglec) that is highly expressed on B cell lymphomas and is a validated target for antibody and nanoparticle-based therapeutics on non-Hodgkin lymphoma (39, 40). Besides, it is reported in some studies that CD22 exerts an important part in lung cancer (41). ALCAM, a 100- to 105-KDa transmembrane immunoglobulin, has been treated as a tumor-specific prognostic marker and demonstrated to take part in activation of T cells, hematopoiesis, angiogenesis, inflammation, and multiple types of tumor propagation and invasiveness (including breast cancer, colorectal cancer, and esophageal cancer) (42–44). CASP1 is the component of the inflammasome that can induce pyroptosis and inhibit angiogenesis and migration of tumor cells (such as lung cancer, breast cancer, and endometrial cancer) (45–47). However, there are few studies on the role of CASP1 in colon cancer. Palmer et al. (48) indicated that CISH, a member of the suppressor of cytokine signaling (SOCS) family, could be induced by TCR stimulation in CD8^+^ T cells and reduce their functional avidity against tumors.

Certain limitations should be noted in the present work. Firstly, more large-scale studies and more experimental methods should be conducted due to the small sample size in this work. Secondly, the present work focused on analyzing the mRNA expression profiles, but it did not consider the associations among lncRNAs, miRNAs, proteins, and other factors; in this regard, more comprehensive studies are needed. Thirdly, there is little research on the role of CD22, CASP1, and CISH in colon cancer, even though it plays an important role. Therefore, more investigations are needed.

To sum up, this study identifies four oxaliplatin resistance-related genes among the colon cancer patients, which are used to construct a signature to predict patient prognosis. According to our results, our constructed four-gene signature can serve as an independent factor to predict the prognosis for colon cancer patients resistant to oxaliplatin. The above results can be used as candidate biomarkers to predict the prognosis for oxaliplatin-resistant colon cancer and shed more light on the theoretical guidance and decision-making for colon cancer clinically.

## Data Availability Statement

The original contributions presented in the study are included in the article/Supplementary Material, further inquiries can be directed to the corresponding author/s.

## Author Contributions

HW and QL conceived and designed the experiments. QL performed the experiments and wrote the paper. LL and HW analyzed the data. All authors have read and approved the final manuscript.

## Conflict of Interest

The authors declare that the research was conducted in the absence of any commercial or financial relationships that could be construed as a potential conflict of interest.

## Supplementary Material

Supplementary Table 1 DEGs between oxaliplatin-resistant and -sensitive colon cancer cells.

Supplementary Table 2 Seventy nine significantly different oxaliplatin resistance-related genes regarding prognosis were discovered.

Supplementary Table 3 The topological properties of the nodes in the PPI network.
